# The Nikki Minaj Effect: The impact of social media disinformation on vaccine hesitancy in the Caribbean

**DOI:** 10.7189/jogh.11.03121

**Published:** 2021-11-13

**Authors:** Sandeep B Maharaj, Darren Dookeeram, Darleen Y Franco

**Affiliations:** 1 Distance Education, Planning and Projects, Faculty of Medical Sciences, The University of the West Indies, St. Augustine Campus, Trinidad and Tobago; 2 Sangre Grande Hospital, Eastern Regional Health Authority, Sangre Grande, Trinidad and Tobago; 3 Primary Care Physician North West Regional Health Authority, Port of Spain Trinidad and Tobago

Members of the Caribbean Community (CARICOM) faced significant challenges in the early days of global vaccination rollout because of various socio-economic-political vulnerabilities [1]. Through skillful diplomatic maneuvering and bilateral agreements, many CARICOM member states were able to secure and procure shipments of the vaccine but are currently reaching plateau levels with respect to their vaccination rollout programmes. **Table 1** below shows the vaccination uptake according to the Caribbean Public Health Agency (CARPHA) [2].

**Table 1:** Vaccination rates in CARICOM states as of 13 September 2021

Country	Percentage (1^st^ dose) vaccination
St Kitts & Nevis	46
Antigua and Barbuda	44
Belize	43
Guyana	42
Barbados	42
Trinidad and Tobago	39
Suriname	36
Dominica	32
Monserrat	29
Bahamas	27
Grenada	23
St. Lucia	20
St Vincent and the Grenadines	17
Jamaica	13
Haiti	0.31

The data demonstrates a variation in COVID-19 vaccine uptake across the Caribbean region that has not yet crossed fifty percent (50%) of any population. While many postulates exist to explain the challenges, ultimately the key drivers (or lack thereof) include public mistrust and misinformation that foster vaccination unwillingness and ultimately hesitancy [3]. This is an ominous finding as some countries are now experiencing third (3rd) and fourth (4th) waves of the pandemic leading to additional profound health and economic burdens [4]. There is currently an overall rising rate of COVID-19 cases and deaths in the region as shown in the figure below [5].

The region finds itself in a precarious position in which the balance between vaccination rollout vs hesitancy is perched at a crossroad that is subject to public perception and vulnerable to rampant disinformation. This carries an inherent adverse potential to the successful coverage of the population against the spread of the disease and by extension suppression of the health burden that COVID-19 carries. It has been well documented that social media platforms have been used for the spread of misleading and unreliable information regarding the pandemic and that unprecedented steps have been taken to curb this newly evolving scourge [6]. Despite these efforts, the so-called “infodemic” regarding the spread of unsupported claims regarding COVID-19 continue and has prompted legal responses in some cases [7]. The recent example of pop star Nikki Minaj, who originates from the Caribbean Island of Trinidad and Tobago, tweeting unsubstantiated information which she linked to the administration of the COVID-19 vaccine demonstrates key points.

“My cousin in Trinidad won’t get the vaccine cuz his friend got it & became impotent. His testicles became swollen. His friend was weeks away from getting married, now the girl called off the wedding. So just pray on it & make sure you’re comfortable with ur decision, not bullied.” *September 13, 2021 [*8*].*

First, at the time of writing we note that Ms Minaj is followed by over 22.8 million other accounts on the social media app Twitter and is considered an influencer because of her large online following and musical ties with the Caribbean region. Whether there was malicious intent or not, it stands to reason that the spread of allegations that to date has been retweeted over 26 000 times with over 150 000 likes, and were subsequently found to be untrue may have dissuaded some persons from becoming vaccinations by stoking fears of impotence as a side effect and was noted by the Ministry of Health to be a colossal waste of time [9].

Second, in addition to the snowballing effect of social media visibility, there have been numerous news stories and time committed to the coverage of this tweet on prominent news media outlets such as the BBC and CNN which may have served to unnecessarily accelerate the growth of the story. The traction gained by the misinformation of the story was so great that questions were being asked of NHS officials in government briefings about the factuality and relevance of the tweet’s claims [9]. On the contrary, there were many tweets, by Ms. Minaj rebuking the misinformation and encouraging persons to do research before getting vaccinated and highlighting issues around mandatory vaccination for work and resumption of economic activity- the media did not focus on any of these to a similar extent.

We wish to make recommendations with regards to such instances moving forward:

1. **Engaging influencers.** The global community, which is closely networked because of advances in technology, requires an elegant transformation of public health education that is sensitive to audience engagement and the susceptibility to influence from those with large followings. It is clear social influencers need to be engaged and placed as a priority group for engagement and education. Influencers who may have views that are divergent from the mainstream pandemic measures, must be presented with the science, research and information in a manner that is transparent and respectful of their views. Additionally, channels should be open for this group to contact experts on issues surrounding the pandemic to ensure they share the best advice.
2. **Encouraging a responsible media.** The media must be cognizant of the embedded messages when selecting stories that may be counterproductive to the public health needs of the global population. There should be efforts to align messages in a manner that reinforces positive behaviours and regales the benefits of public health measures.
3. **Digital innovations to solve digital problems.** The social media platforms should establish and have the capacity to transform themselves and enforce balanced restrictions on public messaging. While it is imperative that the freedom of speech that is enjoyed by many is preserved as an important aspect of democracy, regulatory bodies must be able to cite information that may be dangerous to global public health and restrict its propagation [10]. While this is an enormous task, literature in the digital technology world suggests that Artificial Intelligence and Data Analytics may be effective in achieving such outcomes [11].

**Figure:**
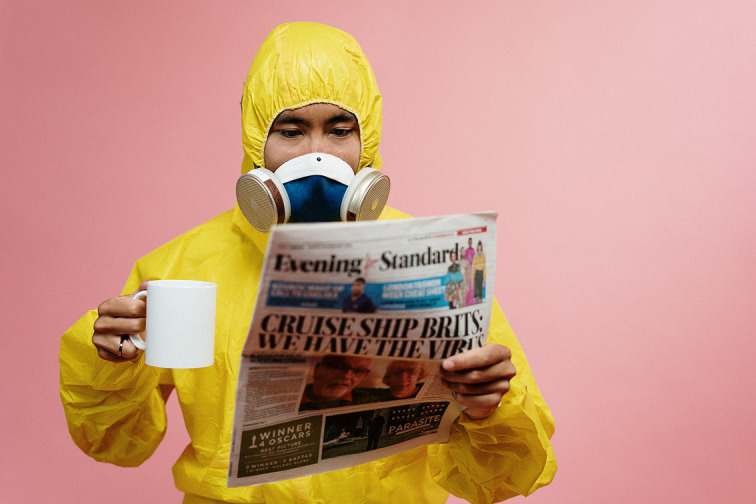
Photo: Ubiquitous media reach – even during a pandemic (from Pexels.com).

The Minister of Health in Trinidad and Tobago has made it publicly clear that there is no evidence to support the claims made by Ms Minaj [12]. It may be more beneficial in achieving the desired outcome of improving vaccination rollout in the Caribbean if she is engaged urgently to inform her position on this matter. Ms Minaj has indicated publicly that she desires more information regarding research and information on the vaccine and this should be provided to positively influence the influencer [13]. The global and Caribbean Media should refocus urgently in an attempt to return the conversation to the topic of the importance of vaccination and safe social practices and away from the irrelevance of the unsubstantiated claims that were made. Finally, there is a plethora of artistes, sports personalities and distinguished academics from many of the islands who are held in high regard within the Caribbean and across the globe who should be actively engaged to share their vaccination stories and personal experiences in dealing with COVID-19. Even though some experts suggest this may not help increase vaccination rates, it will surely expand the message to a wider audience [14]. This issue is a multi-factorial one and therefore requires a multi-level, cross-specialization solution.
